# Recent obstetrical and gynaecological care experiences and their sociodemographic and mental health correlates: A latent class analysis

**DOI:** 10.1177/17455057261455494

**Published:** 2026-06-24

**Authors:** Léa Jeanne Séguin, Sylvie Lévesque, Arianne Jean Thorn, Isabelle Boucoiran, Sarah Landry, Natacha Godbout

**Affiliations:** 1 Department of Sexology, 14845 Université du Québec à Montréal (UQÀM), Montreal, QC, Canada; 2 Department of Obstetrics and Gynecology, 5622 Université de Montréal, Montreal, QC, Canada; 3 Mouvement pour L’autonomie dans L’enfantement, Montreal, QC, Canada

**Keywords:** obstetrical violence, gynaecological violence, provider mistreatment, patient decisional autonomy, medical invalidation, latent class analysis, patient-centred care

## Abstract

**Background:**

High-quality obstetrical and gynaecological care is vital for physical, psychological, and sexual health. However, research has documented widespread patterns of dehumanized and discriminatory care, with marginalized groups disproportionately impacted. Such care can cause lasting harm, underscoring the need for a trauma-informed and person-centred research to better understand and address these experiences.

**Objectives:**

The present study aimed to: (1) identify profiles of recent obstetrical and gynaecological patient-provider interactions in Québec, Canada, and (2) examine their links to sociodemographic characteristics, mental health, and sexual wellbeing.

**Design:**

The current study is cross-sectional.

**Methods:**

A community sample (*n* = 1490) having received gynaecological or obstetric care in the last seven years in Québec completed an online survey assessing sociodemographic characteristics, mental health and sexual wellbeing indicators, as well as provider mistreatment, perceived discrimination, and decisional autonomy during the most recent care experience.

**Results:**

Latent class analyses (LCA) identified a four-class solution. *Humanizing care* (39.7%) featured high decisional autonomy and no mistreatment or perceived discrimination. *Subpar care* (30.7%) showed moderate autonomy with provider neglect, but no discrimination or provider aggression. *Dehumanizing care* (17.7%) and *patronizing care* (11.8%;) involved discrimination and neglect, with the former also facing judgement, aggression, and rights infringements, and the latter, low autonomy but no provider aggression. *Dehumanizing care* class participants were the youngest and most likely to be socially marginalized (e.g., gender- and sexually diverse, racialized, less educated) and to report poor mental health and poor sexual wellbeing. *Humanizing care* participants were predominantly older and cisheterosexual and were the least likely to report poor mental health and sexual wellbeing. *Subpar care* and *patronizing care* classes included more gender- and sexually diverse individuals and intermediate levels of poor mental health and sexual wellbeing.

**Conclusion:**

This study reveals systemic mistreatment in medical care and urges structural reforms, including anti-Black racism training and culturally competent, inclusive practices in gynaecology and obstetrics.

## Plain language summary

Good obstetrical and gynaecological care supports physical, mental, and sexual health, but many people report care that feels dehumanizing or discriminatory, especially those from marginalized groups. Using a trauma-informed, person-centred approach, this study describes recent patient-provider interactions in Québec and examines links with sociodemographic background, mental health, and sexual wellbeing. We surveyed 1,490 people in Québec who recieved obstetrical or gynaecological care within the past seven years. Respondents reported sociodemographics, mental health, sexual wellbeing, and details of their most recent care (mistreatment, perceived discrimination, and decisional autonomy). Latent class analysis identified four groups: (1) Humanizing care (39.7%): high decisional autonomy and no reported mistreatment or discrimination, (2) Subpar care (30.7%): moderate autonomy with provider neglect, but no discrimination or aggression, (3) Dehumanizing care (17.7%): discrimination and neglect plus judgment, aggression, and rights violations, and (4) Patronizing care (11.8%): discrimination and neglect with low autonomy but no aggression. Those in the Dehumanizing care class were youngest, more commonly socially marginalized (e.g., gender- and sexually diverse, racialized, lower education), and most likely to report poor mental health and low sexual wellbeing. Humanizing care participants were more often older and cisheterosexual and reported the best mental health and sexual outcomes. Subpar and Patronizing classes included more gender- and sexually diverse people and had intermediate levels of mental health and sexual wellbeing outcomes. These results reveal systemic mistreatment in obstetrical and gynaecological care and support structural reforms such as anti-Black racism training and culturally competent, inclusive practices to make care safer and more respectful for all.

## Introduction

Access to high quality obstetrical and gynaecological care plays a pivotal role in promoting individuals’ physical, psychological, and sexual health. These healthcare experiences often coincide with some of life’s most vulnerable, yet transformative milestones, such as menstruation, contraception, pregnancy, childbirth, and menopause. When care is respectful, attentive, and centred on patient autonomy, it can foster trust, healing, and sustained engagement with the healthcare system.^1–3^ For example, a systematic review shows that increased patient decisional autonomy or shared decision-making is associated with positive mental health outcomes, including reduced anxiety and depressive symptoms.^4^ Conversely, care that is perceived as disrespectful, coercive, or violent can lead to disengagement, trauma, and deteriorating mental health.^5–8^

In recent years, a growing body of literature has documented the occurrence of negative or dehumanizing care experiences in gynaecology and obstetrics, including reports of judgment, dismissal, physical harm, and infringements on patient rights.^9–13^ A systematic review of studies published between 2014 and 2024 found that the global prevalence of obstetric violence was approximately 59%, with non-consented care being the most common form.^14^ Similarly, a systematic review and meta-analysis of 25 studies published between 2019 and 2023 showed that the overall prevalence of obstetric violence in high-income countries was 45.3%.^15^ In a Spanish cross-sectional study of 899 postpartum women, 67.4% reported experiencing obstetric violence, including 54.5% physical abuse (e.g., manual removal of the placenta without anaesthesia, abdominal compression during pushing, indication of lying down or supine without justification, etc.), 36.7% psycho-affective mistreatment (e.g. preventing companionship, preventing contact with the newborn), and 25.1% verbal abuse (e.g. criticism of the expression of emotions, invalidation, etc.).^16^ Arias Fuentes et al.’s analysis of data from Ecuador’s 2019 National Survey on Family Relations and Gender Violence against Women further supports this trend: among 17,211 women aged 15 and older, approximately one-third (33%) reported experiencing obstetric violence, and two-fifths (42%) reported gynaeco-obstetric violence at least once in their lifetime. These experiences encompassed dehumanizing treatment, abuse of medicalization, and violations of autonomy and decision-making rights.^17^

Complementing these findings, a 2023 study from Cárdenas-Castro and Salinero-Rates (2023) also documented the high prevalence of dehumanizing gynaecological care experiences. In this cross-sectional study conducted with 1,503 women across Chile who had received gynaecological care, about 60% of participants reported experiencing some form of gynaecological violence, such as undergoing unnecessary painful procedures, having their sexual practices judged, or being touched inappropriately given the reason for consultation.^18^ In a large sample of U.S. individuals with gynaecological cancers, the most frequently reported discriminatory experience was not feeling listened to or not being taken seriously by healthcare professionals.^19^

The burden of such experiences does not fall evenly across all populations. Marginalized groups—including racialized individuals, LGBTQ+ people, language minorities, and those with lower socioeconomic status—are disproportionately affected by disrespectful and discriminatory treatment in reproductive healthcare settings.^17,20–23^ In a Chilean sample, higher rates of reported healthcare provider violence were observed among individuals who identify as indigenous (72%), non-heterosexual (71%), of African descent (83%), and who are under 34 years of age (62%).^18^ Similarly, the rates of gynaeco-obstetric violence in Arias Fuentes et al.’s study in Ecuador were significantly higher among women aged 26–35 and 46–55, those with only secondary education, Indigenous and racialized women, and residents of urban areas as well as the Highlands and Amazon regions.^17^ Scholars from feminist, queer, and decolonial traditions have emphasized how structural inequities are embedded in healthcare systems, shaping who receives quality care and who is exposed to harm.^24–27^

The experiences of mistreatment, abuse, disrespect, and violence are not only distressing in the moment, but can also have lasting consequences, including symptoms of anxiety, depression, and post-traumatic stress, as well as reduced sexual wellbeing and avoidance of future care.^5–8,18,28,29^ Moreover, being subjected to more overt forms of provider mistreatment (e.g., verbal, physical, sexual violence) is not necessary to experience negative psychological and sexual outcomes. A growing body of research provides evidence that having experienced medical invalidation^30^ or disenfranchising talk^31^ is sufficient to cause deleterious effects on wellbeing.^32,33^

Despite growing interest in obstetrical and gynaecological mistreatment, most studies do not account for how experiences of care cluster within individuals. Few adopt person-centred methodologies that can identify distinct patient-provider interaction profiles and link them to broader outcomes. Yet, identifying care profiles is essential to move beyond generalized prevalence rates and to better understand how different combinations of care experiences may be associated with psychological health outcomes. Moreover, there is a lack of robust quantitative data from Canada, and from Québec specifically, that speak to the experiences of marginalized populations.

This study aimed to address these gaps by(1) identifying distinct profiles of recent obstetrical and gynaecological patient-provider interactions among individuals in Québec, Canada, and (2) examining how these care profiles were associated with participants’ sociodemographic characteristics, mental health, and sexual wellbeing.

## Method

The present study follows the STROBE guidelines for observational studies (von Elm et al., 2007).^34^ The STROBE checklist is available in the supplementary material.

### Participants and procedure

Of the 2,152 individuals who accessed the online survey as part of the PAROLES action research project, 553 were excluded. These include 522 participants who exited the survey after only providing sociodemographic data, 14 duplicates, 11 nonserious responses, and 6 participants who did not meet the inclusion criteria. The final community sample included 1,490 participants. A minimum of 600 per type of care experience (i.e., gynaecologic and obstetric care) was aimed to have sufficient data for various analyses (for an example, see 35).

Eligible participants were at least 18 years old, were assigned female at birth, and had received gynaecological or obstetric care in the last seven years in Québec. Instead of a five-year criterion, a seven-year criterion was used to account for a potential COVID pandemic effect on care experiences. That said, preliminary analyses (not shown here) suggested no such effect. The cross-sectional data were collected from July 2023 to January 2024.

Participants were recruited primarily through the research team’s and partner and community organizations’ social media pages, as well as through radio and television media outlets. This diversified approach ensured better reach in Québec’s remote areas and difficult to reach populations, increasing the sample’s and care experiences’ representativeness. Participants were entered into a draw for a chance to win one of twenty $50 electronic gift cards. This study was approved by the Université du Québec à Montréal’s Institutional Research Ethics Board (No. 2023-5345) on May 8, 2023. Informed consent was obtained electronically from all subjects involved in the study.

### Measures

#### Sociodemographic variables

Data were collected on participants’ age, sex assigned at birth, gender, sexual orientation, self-reported racial/ethnic minority status, language most spoken at home, birth country and province, educational attainment, and perceived financial situation. For analytical purposes, participants having indicated being female (sex assigned at birth), women (gender) and heterosexual were categorized as cisheterosexual. All other participants were categorized as gender- and sexually diverse.

#### Patient-provider interaction indicators during most recent gynaecologic/obstetric care experience

##### Patient decisional autonomy

The 7-item Mother’s Autonomy in Decision Making Scale (MADM) was used to measure the degree to which patients were involved in the decision-making, were informed about their options and had sufficient time to consider them, and their choices were respected by their healthcare providers.^36^ The original items pertained to obstetrical care. Thus, for the present study, the items were adapted to assess decisional autonomy in both obstetrical and gynaecological care by removing references to babies and childbirth (e.g., “The provider told me there are several treatment or intervention options for my problem”; “I was able to choose the care options that seemed best to me”). While the original scale has been validated, this adapted version was not. Answers ranged from *1–Strongly disagree* to *6–Strongly agree*. Scores were added to create total MADM scores, which were then recoded according to tertiles: scores ranging from 6 to 17 were recoded as low autonomy,^1^ those ranging from 18 to 29 were recoded as moderate autonomy,^2^ and those ranging from 30 to 42, as high autonomy.^3^ In the present sample, internal consistency was excellent (α = .95).

##### Mistreatment

The 14-item Pregnant Persons Experience of Mistreatment by Providers Index (MIST) was used to measure experiences of mistreatment during participants’ last care experience. These items were developed based on the Giving Voices to Mothers study.^37^ Responses ranged from *1–Never to 5–All the time*. An exploratory factor analysis was performed to determine whether distinct forms of mistreatment could be explained by the data, and therefore, whether different mistreatment indicators should be created, as they could increase latent class interpretability. The data were analysed using maximum likelihood factor analysis with oblimin rotation. Analyses suggested a 3-factor solution accounting 62.08 of the variance. This solution was considered adequate because it had no non-loading or cross-loading items,^38^ all sufficient loadings (≥.40),^38^ no single- or two-item factors,^39^ its factors were interpretable^40^ and all factors had eigenvalues greater than 1.^41^

The three factors were named: provider neglect (3 items; e.g., “The health professionals ignored me or talked about me as if I wasn’t there”), provider violence (6 items; e.g., “I was the victim of physical violence [such as aggressive physical contact, unnecessary painful acts, etc.]”), and patient right infringement (5 items; e.g., “My confidential or personal information has been disclosed without my consent”). A closer inspection of provider violence items suggested that they represented two types of violence. Thus, this factor was divided into two mistreatment indicators: provider judgement (3 items; e.g., “The healthcare provider told me I already had too many children”) and provider aggression (3 items; e.g., “I was threatened by the healthcare provider”). Scores on each item were dichotomized (*0–No; 1–Yes*) and added to create total scores for each mistreatment indicator. Then, for each indicator, scores were recoded as 0–No mistreatment, 1–One form of neglect/judgement/aggression/one right infringed, and 2–More than one form of neglect/judgement/aggression/one right infringed. Cronbach’s alphas were .83 for provider neglect, .77 for provider judgement, .83 for provider aggression, and .77 for patient rights infringement.

##### Perceived discrimination

Perceived discrimination during the most recent care experience was assessed using the single item “During your last consultation, did you feel you were treated less well than other people for various reasons?” (*0–No; 1–Yes*).

#### Outcome variables

Four wellbeing indicators were assessed. First, participants were asked whether they had consulted a health professional about their emotional or mental health in the past 12 months (*0–No; 1–Yes*) and, if so, whether the reason for this consultation was related to their most recent obstetric or gynaecological care experience (*0–No; 1–Yes*). Participants were categorized into one of two categories: those who had not consulted for their emotional or mental health or who had done so but for a reason unrelated to their most recent care experience (0), and those who had consulted for a reason related to their most recent care experience.^1^

Second, the 5-item Primary Care PTSD screen (PC-PTSD) was used to measure posttraumatic stress symptoms (e.g., “Following your care AND for at least 1 month… did you have nightmares about this event or even think about it without wanting to think about it?”; “… did you feel detached from others, activities, or those around you?”).^42^ Answers to each statement, which were dichotomous (*0–No; 1–Yes*), were added to create total PC-PTSD scores. However, given that scores were severely positively skewed, participants were categorized into one of three categories: those who reported no posttraumatic stress symptoms (0), those who reported one to two symptoms (1), and those who reported three to five symptoms (2).

Third, debilitating psychological symptoms were assessed using three items (i.e., “Following the last obstetrical or gynaecological care I received… I experienced anxiety/sadness/sleep disturbances to such a degree that I had difficulty performing my daily tasks”). Responses ranged from *0–No to 3–Often*. These items are adapted from national epidemiological surveys to assess the presence of core symptoms of anxiety, depression/sadness, and sleep disturbances, as well as their functional impact over a specified period.^43^ Participants’ scores were added to create total debilitating psychological symptoms scores. Due to significant positive skewness, participants were categorized as follows: those with a score of 0 (0), those with a score of 1 to 3 (1), those with a score of 4 to 6 (2), and those with a score of 7 to 9 (3). In the present sample, internal consistency for this indicator was excellent (α = .90).

Fourth, the most recent care experience’s impact on participants’ overall sex life quality was measured using a single item (i.e., “The last obstetrical or gynaecological care I received, as described in the previous sections, had a negative impact on the quality of my sex life [e.g., erotic touching, masturbation, sexual play, intercourse]”). Answers, which ranged from *1–Strongly disagree to 6–Strongly agree*, were then dichotomized (*0–No; 1–Yes*).

### Statistical analysis

To identify types of patient-provider interactions, a latent class analysis (LCA) was performed using LatentGOLD 6.1.^44^ LCA uses observed indicators to detect latent (or unobserved) heterogeneity in samples, or “classes”.^45^ Six indicators were included to describe patient-provider interactions during participants’ most recent gynaecologic/obstetric care experience: (1) patient decisional autonomy, (2) provider neglect or dismissal, (3) provider judgement, (4) provider aggression, (5) patient rights infringement, and (6) perceived discrimination. In light of the empirical literature, we expected analyses to produce at least three classes: one class characterized by overall positive patient-provider interactions (absence of mistreatment and perceived discrimination, and high autonomy), another by overall negative experiences (high frequencies of mistreatment, perceived discrimination, and low autonomy), and another by high frequencies of provider neglect or dismissal and low autonomy, but low levels of provider aggression, judgement, and rights infringement.

First, LCA models including 1 to 6 classes were estimated. The models were compared across fit indices and class sizes. Low Bayesian Information Criterion (BIC) and bootstrap L^2^ *p*-values greater than .05 suggest better model fit, and entropy values closer to 1 indicate better class separation.^46^ While the L^2^ *p*-value is traditionally considered when determining model fit, it can be overly sensitive when using a large number of class indicators, as this increases model complexity, causes the data to become sparse, and disrupts χ^2^ distribution,^47^ leading to the rejection of well-fitting models. Thus, the bootstrap L^2^ *p*-value, which is more robust in such cases,^48^ has been prioritized during model selection. Bivariate residuals of a value of four or less also indicate better model fit, as they indicate that there is negligeable residual association remaining that is unexplained by the classes.^47^ Lastly, class interpretability was also considered.^49^ Wald tests for paired comparisons were used to assess class differences on class indicators. Based on posterior probabilities, participants were assigned to latent classes.

To assess class differences on sociodemographic variables and mental health and sexual wellbeing outcomes, we performed between-class ANOVA-type comparisons implemented in LatentGOLD using the Bolck-Croon-Hagenaars (BCH) modified bias-correction method, which accounts for uncertainty in class membership.^50,51^ The modified BCH approach has been recommended for both continuous and binary outcomes variables.^51^ At each step, we computed robust variance estimations to prevent standard errors underestimation. When omnibus tests utilizing Wald’s statistic revealed significant between-class differences, we examined the Bonferroni-corrected post hoc comparisons to maintain the familywise Type I error rate at 0.05 (by dividing the Type 1 error rate by the number of pairwise comparisons). When conducting class comparisons on sociodemographic variables and mental health and sexual wellbeing outcomes, missing data were handled using Full Information Maximum Likelihood (FIML) estimation.

## Results

### Participants

Most participants were between 26 and 35 years old (50.7%), born in Canada (89.7%) and Quebec (87.4%), and identified as cisgender women (93.8%) and heterosexual (82.1%). Most of the sample reported French as the language most spoken at home (82.6%), followed by English (15.6%). Most participants (63.4%) had at least an undergraduate degree and reported having sufficient income to meet basic personal or family needs (63.2%). One-fifth of participants received their care in Montreal (21.6%), followed by Montérégie (10.9%) and the Capitale-Nationale (9.7%). Of the 1,490 participants, 908 responded to the gynaecological care section and 651 responded to the obstetrical care section, thus totalling 1,599 recent care experiences. Table 1 presents the sample’s characteristics.

**Table 1.** Sample characteristics (*n* = 1490).

Characteristic	*n* ^a^	%^b^
Age	**1462**	​
18–25	165	11.1
26–35	755	50.7
36–45	358	24.0
46–55	124	8.3
56 and over	88	5.9
Gender	**1484**	​
Cisgender women	1397	93.8
Gender diversity^c^	87	6.2
Sexual orientation	**1476**	​
Heterosexual	1212	82.1
Sexual diversity^d^	264	17.9
Race/ethnicity	**1490**	​
White	1205	80.9
Racialized	285	19.1
Place of birth	**1482**	​
Canada	1329	89.7
Québec	1159	87.4
Outside Québec	167	12.6
Outside Canada	153	10.3
France	75	54.7
United States	9	6.6
China	8	5.8
Other	45	32.9
Language spoken at home	**1483**	​
French	1231	82.6
English	232	15.6
Other	20	1.4
Highest educational attainment	**1484**	​
High school diploma or less	99	6.8
College, vocational or trade school	440	29.6
University degree	945	63.6
Perceived financial situation	**1482**	​
More than sufficient income (to meet basic personal or family needs)	420	28.3
Sufficient income	941	63.5
Insufficient income	121	8.2
Recent gynaecological care experience	908	57.0
Recent obstetrical care experience	691	43.0

Bold indicates the total valid n for each variable.

^a^*Notes.* *n* may not total 1,490 due to missing data.

^b^These are valid percentages (i.e., they exclude missing cases).

^c^Includes all individuals who reported a gender that did not match their sex assigned at birth.

^d^Includes homosexual/gay/lesbian, bisexual, pansexual, queer, asexual, and questioning individuals.

### Model selection

A four-class solution was identified as the optimal model (see Table 2). While model 3 has a high entropy value (>.80) and is the first model to have an L^2^ *p*-value greater than .05, its maximum bivariate residual is much higher than the conventional cutoff value of four, and its significant bootstrap L^2^ *p*-value indicates poor model fit. Further, while model 5 is the first model to have a bootstrap L^2^ *p*-value greater than .05 and has a very low maximum bivariate residual, it has a lower entropy value than model 4 and some of its classes were conceptually indistinct from one another. Thus, model 4 was selected because it had the lowest BIC, a maximum bivariate residual close to the conventional cutoff value, a high entropy value, and its classes were interpretable as conceptually distinct.

**Table 2.** Goodness of fit indices of LCA models.

Number of classes	LL	N of parameters	BIC	AIC	L^2^ *p*-value	Bootstrap L^2^ *p*-value	Max. BVR	Entropy
1	-4980.97	11	10038.18	9983.93	*<* 0.001	*<* 0.001	575.20	1
2	-4182.14	18	8489.05	8400.28	*<* 0.001	*<* 0.001	54.52	0.85
3	-4009.14	25	8191.57	8068.29	0.97	*<* 0.001	17.12	0.84
**4**	**-3967.73**	**32**	**8157.26**	**7999.46**	**1.00**	**0.01**	**4.13**	**0.79**
5	-3947.63	39	8165.58	7973.26	1.00	0.11	1.44	0.77
6	-3934.45	46	8187.76	7960.91	1.00	0.38	1.65	0.75

The model in bold is the selected model.

### Latent classes

The indicators’ conditional probabilities for each class are presented in Table 3. Class 1 (39.70%; humanizing care) included participants whose most recent care experience was characterized by high levels of patient decisional autonomy, no perceived discrimination, no experiences of provider neglect, judgement, and aggression, and no patient rights infringed. Class 2 (30.75%; subpar care) was composed of participants who reported moderate levels of patient decisional autonomy, no perceived discrimination, no patient rights infringed, and no experiences of provider judgement and aggression, but more than one form of provider neglect during their most recent care experience. Class 3 (17.70%; dehumanizing care) included participants whose most recent care experience was characterized by perceived discrimination, at least one patient right infringed, and more than one form of provider neglect, judgement, and aggression, but moderate levels of patient decisional autonomy. Class 4 (11.85%; patronizing care) described participants who mostly experienced no provider judgement and aggression and who had no patient rights infringed, but more than one form of provider neglect, perceived discrimination, and low levels of patient decisional autonomy during their most recent care experience.

**Table 3.** Class description across patient-provider interaction indicators.

Size (%)	Total sample	Class 1	Class 2	Class 3	Class 4	Between-class differences
		Humanizing care	Subpar care	Dehumanizing care	Patronizing care	
		39.70%	30,75%	17.70%	11,85%	
**Patient decisional autonomy (%)**						1,2***; 1,3***; 1,4***
Low autonomy^6-17^	13.68	0.30	8.77	10.78	**75.48**	​
Moderate autonomy^18-29^	31.13	12.55	**47.63**	**50.06**	23.30	​
High autonomy^30-42^	55.19	**87.14**	43.60	39.16	1.21	​
**Perceived discrimination**						1,2*; 1,3***; 1,4***; 2,3***; 2,4**
No	79.05	**99.31**	**89.64**	41.04	39.27	​
Yes	20.95	0.69	10.36	**58.96**	**60.73**	​
**Neglect**						1,2***; 1,3***; 1,4***; 2,3***; 2,4*
No neglect	36.08	**82.15**	10.60	0.23	0.17	​
One form of neglect	14.16	14.62	23.69	4.21	3.59	​
More than one form of neglect	49.76	3.23	**65.71**	**95.56**	**96.24**	​
**Judgement**						1,2***; 1,3***; 1,4***; 2,3**; 2,4*; 3,4***
No judgement	66.25	**97.56**	**70.84**	0.02	**47.22**	​
One judgement	12.28	2.41	24.08	1.58	35.87	​
More than one judgement	20.95	0.03	5.08	**98.41**	16.91	​
**Aggression**						1,2**; 1,3***; 1,4***; 2,3***; 2,4**; 3,4**
No aggression	64.45	**98.83**	**67.50**	0.04	**37.57**	​
One form of aggression	13.09	1.16	25.55	2.38	36.69	​
More than one form of aggression	22.46	0.01	6.95	**97.58**	25.74	​
**Right infringement**						1,2**; 1,3***; 1,4***; 2,3***; 3,4***
No rights infringed	79.59	**96.12**	**85.02**	**37.37**	**73.18**	​
One right infringed	12.50	3.66	12.22	28.46	19.02	​
More than one right infringed	7.91	2.22	2.77	34.17	7.80	​

*Notes.* Wald tests for paired comparisons were used to assess class differences on class indicators. Differences between classes are significantly different at *p<* .05*, *p<* .01**, *p<* .001*** (Bonferroni corrected). Percentages in bold represent the most distinctive features of each class.

Participants excluded from LCA analyses (31.27 %) due to significant missing data on care experience indicators were more likely to be white, χ^2^ (1, *n* = 1490) = 23.52, *p* < 0,001, φ_c_ = .126, born in Quebec, χ^2^ (1, *n* = 1326) = 8.63, *p* = 0,003, φ_c_ = .081, and to be French-speaking, χ^2^ (1, *n* = 1483) = 28,98, *p* < 0,001, φ_c_ = .140. Participants with missing data did not significantly differ from those with complete data regarding age, gender and sexual orientation, country of birth, education, and self-perceived financial situation.

### Class differences on sociodemographic characteristics

Table 4 shows the four latent classes’ sociodemographic composition. Bonferroni-corrected pairwise comparisons revealed that participants in the *dehumanizing care* class were significantly younger (*M =* 30.22 years) than those in all other classes, and participants in the *humanizing care* (*M =* 36.40 years) and *patronizing care* (*M =* 37.74 years) classes were older than those in both the *subpar care* (*M =* 33.70 years) and *dehumanizing care* groups. Cisheterosexual individuals were overrepresented in the *humanizing care* class (89.14%), while gender- and sexually diverse individuals were overrepresented in the *dehumanizing care* group (36.98%). The *subpar care* (21.06%) and *patronizing care* (21.40%) classes were also more likely than the *humanizing care* class to include gender- and sexually diverse participants. Analyses also showed that racialized participants and language minorities were overrepresented in the *dehumanizing care* class (81.76% and 76.04%, respectively). Participants in the *dehumanizing care* class were also more likely than those in all other classes to only have a high school diploma or less (17.13%), and less likely than those in the *humanized care* class to have more than sufficient income (21.01 vs. 32.31%). *Dehumanizing care* class participants were also more likely to have been born outside of Quebec (53.64%) than were those in all other classes.

**Table 4.** Sociodemographic composition of latent classes.

Sociodemographic variables	Total sample	Class 1	Class 2	Class 3	Class 4	*p* value	Between-class differences
		Humanizing care	Subpar care	Dehumanizing care	Patronizing care		
**Age *M* (*SE*)**	34.64 (0.28)	36.40 (0.48)	33.70 (0.52)	30.22 (0.64)	37.74 (1.29)	**< .001**	1,2**; 1,3***; 2,3***; 2,4*; 3,4***
**Sexual orientation/gender (%)**						**< .001**	1,2*; 1,3***; 1,4*; 2,3**
Sexual/gender diversity	19.83	10.86	21.06	36.98	21.40	​	​
Cisheterosexual	80.17	89.14	78.94	63.02	78.60	​	​
**Race (%)**						**< .001**	1,3**; 2,3***; 3,4**
Racialized	22.46	13.02	7.64	81.76	3.99	​	​
White	77.54	86.98	92.36	18.24	96.01	​	​
**Language (%)**						**< .001**	1,3***; 2,3***
Language minority^a^	20.50	10.56	9.70	76.04	0	​	​
French	79.50	89.44	90.70	23.96	100	​	​
**Education (%)**						**< .001**	1,3**; 2,3**; 3,4*
High school diploma or less	7.34	7.55	1.96	17.13	6.01	​	​
College/vocational/trade school	29.82	31.10	26.13	35.94	25.97	​	​
University degree	62.84	61.35	71.91	46.93	68.03	​	​
**Perceived financial situation (%)**						**.05**	1,3*
Insufficient income	8.30	7.86	9.19	4.48	13.21	​	​
Sufficient income	63.65	59.82	61.38	74.51	66.12	​	​
More than sufficient income	28.05	32.31	29.43	21.01	20.67	​	​
**Place of birth**						**< .001**	1,3***; 2,3***; 3,4***
Québec	77.18	80.98	84.26	46.36	92.07	​	​
Other province/territory/country	22.82	19.02	15.74	53.64	7.93	​	​

*Note*. Differences between classes are significantly different at *p<* .05*, *p<* .01**, *p<* .001*** (Bonferroni corrected). *M* = mean; *SE* = standard error. All estimates are measurement-error weighted based on the BCH approach in LatentGOLD 6.1.

^a^Includes all languages other than French.

### Class differences on mental health and sexual wellbeing

Table 5 shows between-class differences on mental health and sexual wellbeing. Participants in the *humanizing care* class were significantly less likely than those in all other classes to have had a mental health consultation in the last 12 months for a reason related to their last care experience (2.02%). Conversely, individuals in the *dehumanizing care* class (51.53%) were significantly more likely to have done so than participants of all other groups. No differences were found between the *subpar care* (9.15%) and *patronizing care* (14.15%) classes on this variable.

**Table 5.** Class differences on mental health and sexual wellbeing outcomes.

Outcome variables	Total sample	Class 1	Class 2	Class 3	Class 4	*p* value	Between-class differences
		Humanizing care	Subpar care	Dehumanizing care	Patronizing care		
**Mental health consultation for a reason related to the last care experience (%)**						**< .001**	1,2*; 1,3**; 1,4***; 2,3***; 3,4***
No	85.55	97.98	90.85	48.47	85.49	​	​
Yes	14.45	2.02	9.15	51.53	14.51	​	​
**Negative impact on sexual well-being (%)**						**< .001**	1,2***; 1,3***; 1,4***; 2,3***; 2,4***
No	72.49	94.73	75.49	33.78	47.85	​	​
Yes	27.51	5.27	24.51	66.22	52.15	​	​
**Post-traumatic stress symptoms (%)**						**< .001**	1,2***; 1,3***; 1,4***; 2,3***; 2,4**; 3,4**
0	54.40	75.84	55.90	16.30	35.47	​	​
1-2	22.46	17.58	25.42	24.44	28.22	​	​
3-5	23.14	6.59	18.68	59.26	36.31	​	​
**Debilitating psychological symptom frequency (%)**						**< .001**	1,2***; 1,3***; 1,4***; 2,3***; 3,4***
0	50.79	74.18	51.74	6.89	35.47	​	​
1-3	17.60	16.40	21.62	10.67	21.53	​	​
4-6	19.09	7.97	19.84	36.30	28.70	​	​
7-9	12.52	1.45	6.80	46.14	14.30	​	​

*Note*. Differences between classes are significantly different at *p<* .05*, *p<* .01**, *p<* .001*** (Bonferroni corrected). *M* = mean; *SE* = standard error. All estimates are measurement-error weighted based on the BCH approach in LatentGOLD 6.1.

Similarly, individuals in the *humanizing care* class (5.27%) were significantly less likely than those in all other classes to report that their most recent care experience negatively impacted their sexual wellbeing, and those in the *dehumanizing care* (66.22%) and *patronizing care* (52.15%) classes, the most likely. Participants in the *patronizing care* class were also significantly more likely than those in the *subpar care* class (24.51%) to indicate that their sexual wellbeing was negatively influenced by their last care experience.

*Humanizing care* class participants reported the lowest levels of post-traumatic stress symptoms (24.17% reported at least one symptom), followed by those in *the subpar care* class (44.10% reported at least one symptom), those in the *patronizing care* class (64.54% reported at least one symptom), and those in the *dehumanizing care* class (83,70% reported at least one symptom). Individuals in the *dehumanizing care* class reported higher levels of post-traumatic stress symptoms than those in all other classes. *Humanizing care* class participants also reported the lowest levels of debilitating psychological symptoms following their most recent obstetrical or gynaecological care experience (25.88% reported at least one symptom), and those in the *dehumanizing care* group (93.11% reported at least one symptom), the highest levels. However, individuals in the *subpar care* group (48.26% reported at least one symptom) did not significantly differ from those in the *patronizing care* group (64.53% reported at least one symptom) on psychological symptoms.

## Discussion

The present study pursued two primary objectives: (1) to identify distinct profiles of recent obstetrical and gynaecological care experiences among individuals in Québec, Canada, and (2) to examine the associations between these care profiles and participants’ sociodemographic characteristics, mental health, and sexual well-being. Diverging from prior research, this study employed a person-centred approach to elucidate patterns of mistreatment and their psychological consequences within a Canadian context.

Within the current sample, provider neglect emerged as the most common form of medical mistreatment (63.9%), surpassing reported rates of provider aggression (35.5%) and provider judgment (33.2%), consistent with some previous empirical evidence. For example, in a large sample of U.S. individuals with gynaecological cancers, the most frequently reported discriminatory experience was not feeling listened to by healthcare professionals, followed by perceptions of being regarded as unintelligent, receiving less courtesy or respect than others, and obtaining substandard services.^19^

The observed association between increasing rates of provider neglect and diminished patient decisional autonomy is conceptually coherent, as fostering patient autonomy inherently requires active inclusion of the patient in clinical discussions. By contrast, neglect inherently excludes patients from these critical conversations. This finding is congruent with prior research linking medical invalidation or disenfranchising talk to reduced patient autonomy and heightened feelings of powerlessness.^33,52–54^ For example, patients who have experienced disenfranchising talk frequently report sensations of bodily alienation and perceive decision-making regarding diagnostic testing and treatment as largely beyond their control.^33^

Sociodemographic disparities were observed across patient-provider interaction classes, with non-French native speakers, racialized and gender- and sexually diverse individuals notably underrepresented in the *humanized care* class and overrepresented in the *dehumanizing care* class. This pattern is consistent with extant literature documenting that these populations face higher rates of healthcare provider discrimination and mistreatment compared to their hegemonic language-speaking,^55^ white,^53,56–58^ and cisheterosexual counterparts,^58^ particularly among immigrant^53^ and transgender patients.^59^

Regarding racialized groups, qualitative research involving Black women in Canada who were pregnant or had given birth within the past three years reveals experiences of dehumanizing, low-quality care characterized by dismissal of concerns, disbelief, neglect, and objectification.^60,61^ The minimization or disregard of symptoms and health concerns is disproportionately reported not only by women relative to men^62^ but also by racialized patients compared to their white counterparts.^63^ At the intersection of race and gender, racialized women may be particularly vulnerable to such treatment. For instance, some healthcare providers avoid discussing gynaecological symptoms with patients due to assumptions that these patients are embarrassed or perceive such symptoms as normal—biases predominantly directed toward racialized or minority ethnic patients.^64^ Moreover, racialized women exhibit a heightened risk of discrimination in maternity care relative to white women,^65^ which may partly reflect their increased susceptibility to pregnancy-related medical conditions such as diabetes and hypertension,^66^ conditions that have been linked to greater healthcare discrimination.^65^ Concerning gender- and sexually diverse populations, these findings corroborate prior studies indicating that such individuals generally,^58,67,68^ and sexually diverse women specifically,^69,70^ frequently avoid or delay seeking care due to fears of stigmatization.

The elevated rates of perceived discrimination observed among the youngest (*dehumanized care*) and oldest (*patronizing care*) groups replicates previous research findings^17,71^ and may be attributable to distinct manifestations of age bias. Research indicates that young women are more likely to encounter healthcare provider refusal for procedures with permanent fertility implications (e.g., hysterectomy, tubal ligation)^31^ and are often perceived as less susceptible to serious pathologies such as endometriosis or gynaecological cancers, resulting in reduced specialist referrals.^64^ Conversely, complaints and symptoms reported by older women are frequently ascribed to “just old age”^72^ or normalized as inherent aspects of menopause or perimenopause,^73^ thereby increasing the likelihood of their concerns being dismissed or minimized. Moreover, perceived discrimination among older participants could be attributable to the fact that being an older expectant mother remains a stigmatized social identity.^74^

Elevated levels of perceived discrimination were documented not only within the *dehumanized care* class—characterized by the overrepresentation of socially marginalized groups—but also among the predominantly white, highly educated, Quebec-born, French-speaking majority comprising the *patronizing care* class. This may be explained by the possibility that, in some cases, such experiences may be arbitrary, with patients encountering healthcare professionals who may be less empathetic, more dismissive, or inadequately trained in patient-centred communication, regardless of the patient’s personal characteristics. It is also plausible that perceived discrimination may be associated with social categories or factors not included in the present analyses, such as body size (e.g., fat individuals), disability status, or specific reasons for consultation (e.g., chronic gynaecological pain, gestational diabetes, cervical cancer). For instance, a systematic review of 25 studies published between 1998 and 2023 found that fat women seeking gynaecological care are often informed that their weight may be the underlying cause of their symptoms and must be addressed prior to investigating alternative causes or treatment options,^64^ effectively delaying or obstructing access to appropriate care. Similarly, according to the same review, women with chronic gynaecological conditions—such as endometriosis and chronic pelvic pain—frequently experience dismissal of their symptoms and concerns by primary care providers, who attribute these issues to psychological factors (e.g., depression or anxiety) rather than physiological causes. Research further indicates that gender intersects with chronic pain perceptions, wherein female patients are more likely to be perceived by healthcare providers as less credible, experiencing less pain, or exaggerating their symptoms.^75^ This bias results in reduced provision of adequate pain management and a greater propensity to recommend psychotherapy over pharmacological interventions.^75^ In such contexts, the minimization or dismissal of patients’ symptoms may be interpreted as discriminatory experiences irrespective of healthcare providers’ intentions.

The present analyses further demonstrated a positive association between the severity of patient mistreatment and posttraumatic stress symptoms. Individuals classified within the *dehumanizing care* group reported the highest levels of such symptoms, followed sequentially by those in the *patronizing care*, *subpar care*, and *humanized care* groups. Notably, even participants who experienced solely provider neglect (*subpar care*) exhibited moderate levels of posttraumatic stress and debilitating psychological symptoms, underscoring that medical neglect alone can impair mental health. While extensive research has documented the detrimental effects of healthcare provider violence—including obstetric violence—on patient mental health,^7,28,76^ an emerging body of literature indicates that medical invalidation per se is sufficient to precipitate adverse wellbeing outcomes.^32,33,77–79^ For example, a meta-synthesis encompassing 151 studies and 11,307 participants revealed that symptom invalidation engenders feelings of shame and suicidality, as well as healthcare-related anxiety, trauma, avoidance, and diagnostic delays.^32^ Complementing these findings, an analysis of narratives from 399 women with chronic overlapping pain conditions across 22 countries indicated that disenfranchising communication not only undermines the patient-provider relationship and perceptions of healthcare systems, but also elicits profound emotional distress, including suicidality in some cases.^31^ According to the same analysis, silencing behaviours—characterized by providers acting as gatekeepers who terminate or limit access to further care (e.g., stating “we have done all we can do” or “there is nothing else we can do for you”) or by rushing appointments thereby discouraging patients from discussing their symptoms were particularly deleterious to patients’ mental health.

The present study additionally identified a significant association between reduced patient decisional autonomy and heightened posttraumatic stress and debilitating psychological symptoms. Supporting this finding, a multi-level meta-analysis of 34 studies conducted between 1990 and 2021 demonstrated that individuals who experienced lower levels of effective decision-making during intrapartum care—characterized by active involvement in decision processes, respect for personal preferences, and a sense of control—reported increased posttraumatic stress symptoms.^80^ Similarly, a systematic review and meta-analysis of 10 studies comprising 4,995 participants found that individuals undergoing emergency caesarean sections were significantly more likely to develop postpartum posttraumatic stress disorder compared to those receiving elective caesarean sections.^81^

Most participants within the *humanizing care* group reported an absence of posttraumatic stress and debilitating psychological symptoms, as well as no adverse effects on their sexual wellbeing. This finding suggests that a care context characterized by the promotion of patient decisional autonomy alongside the absence of mistreatment fosters psychological wellbeing. Nonetheless, approximately one-quarter of individuals in this group reported experiencing at least one posttraumatic stress symptom and a nonzero frequency of debilitating psychological symptoms. This variability may be attributable to the reason for consultation—for example, routine Pap smear or standard monitoring of a healthy pregnancy may be less stressful experiences than an evaluation for suspected cancer or endometriosis or care following a miscarriage. Additionally, the intimate and invasive nature of gynaecological and obstetric care may elicit negative emotional responses in women who have experienced sexual violence,^82^ even in contexts of respectful care. Lastly, given that the present study only assessed participants’ most recent care experience, it is possible that some individuals classified within the *humanized care* class may have previously encountered other, more adverse care experiences, potentially contributing to complex posttraumatic stress disorder symptomatology.

The present study also found the severity of mistreatment to be significantly associated with sexual distress, with individuals in the *dehumanized care* and *patronizing care* classes most likely to report that their most recent care experience adversely affected their sexual wellbeing, followed by those in the *subpar care* class. This escalation in poor sexual wellbeing may not only reflect the higher incidence of mistreatment within these groups but may also be influenced by the reason for consultation. Adverse gynaecological consultations, particularly those involving a gynaecological cancer diagnosis or treatment, for example, have been shown to substantially impair sexual wellbeing.^83,84^ These effects are multifaceted, encompassing both physical and psychological domains, including treatment-related sequelae and alterations in body image and self-esteem.^85,86^ Furthermore, negative or traumatic obstetrical care experiences have been linked to diminished sexual function and wellbeing.^87–89^

### Limitations

The present findings should be interpreted considering several limitations. First, the present sample was primarily cisgender, heterosexual, white, French-speaking, highly educated, and identified as women. Thus, the findings may not be generalizable to marginalized groups. In addition, the class indicators included in the analyses were predominantly “negative” (i.e., mistreatment and perceived discrimination), with patient decisional autonomy as the sole “positive” indicator, thereby neglecting other dimensions of quality patient-provider interactions. Although a measure of respectful treatment was initially incorporated alongside decisional autonomy, model instability and elevated bivariate residuals necessitated its exclusion, resulting in the retention of only one “positive” interaction indicator. Future latent class analysis research should incorporate additional indicators of interaction quality, such as provider empathy, patient satisfaction, and trust in the healthcare system. Also, while the patient autonomy measure was adapted from a validated scale originally developed for obstetrical care, its use in a broader context including gynaecological care has not been formally validated. As such, the psychometric properties of the adapted measure in this context remain uncertain, and findings should be interpreted with caution.

Furthermore, the analyses did not consider clinical or contextual variables that may influence patient-provider interaction quality, including healthcare professional type (e.g., nurse, general practitioner, midwife, specialist), provider gender, or care setting (e.g., private versus public; hospital versus walk-in clinic). Additionally, gynaecological and obstetrical care experiences were aggregated without differentiation; separate analyses may reveal distinct patient-provider interaction quality classes specific to each care domain.

The study is also susceptible to selection bias, as individuals with negative care experiences may have been more motivated to participate than those with positive or satisfactory experiences. Consequently, the identified patient-provider interaction classes and their relative sizes may overrepresent adverse care experiences than what may be observed in actual practice. Having participants report about their most recent care experience as opposed to their care experiences overall may have helped to control for this bias in addition to recall bias. Moreover, in the absence of population data, the present findings provide some insight into gynaecological and obstetrical care experiences in Québec. In addition, because no a priori power analysis was conducted for the latent class analysis, the confidence in the adequacy of the sample size may be limited, particularly with respect to detecting small classes and ensuring the stability of the selected class solution. Finally, the cross-sectional design precludes causal inference regarding the observed associations. For example, while it is plausible that individuals classified within the *dehumanized care* class were more likely to seek help for mental health-related reasons due to their last care experience, it is equally possible that these individuals had preexisting tendencies to engage with mental health services due to factors such as prior awareness or previous consultations. Longitudinal research is warranted to elucidate the directionality and causality of these relationships more definitively. Future research should also incorporate qualitative approaches to better contextualize and understand experiences of mistreatment that may not always be adequately captured through survey-based measures.

### Implications

The present findings suggest that the quality of care should not only be evaluated based on standardized protocols and biomedical appraisals of patients’ health issues, but also on the notion of patient-centeredness, or the extent to which the provider solicits the patient’s perspective, considers their psychosocial context, comes to a joint understanding of the health issue and treatment with the patient, and involves the patient in decision-making. As the present study suggests, medical neglect is by far the most common form of provider mistreatment. Addressing this issue may necessitate structural reforms, such as implementing comprehensive bias training, enhancing communication protocols, and establishing institutional accountability mechanisms. For instance, patient welfare may benefit from the systemic monitoring of practices via anonymous questionnaires and participatory audits, and increased restorative or preventive interventions.

Fostering provider empathy through formalized training and continued education may be an additional solution. Provider empathy has been found to be associated with many positive patient outcomes such as reduced anxiety^90,91^ and depression,^92,93^ increased trust in physician’s competence, and improved physician-patient relationship.^94^ Physician empathy has also been found to deliver significantly better clinical outcomes.^95,96^ Some evidence suggests that patients’ ratings of their provider’s ability to explain information clearly, listen carefully, provide understandable information, and show respect are significantly correlated with physician empathy scores.^97^ In another study, positive patient-provider interactions—those that are characterized by provider empathy—have been described by women patients as having the existence, abnormality, and nature of their symptoms affirmed, even in the absence of affirmative test results, being listened to and asked questions, being given the opportunity to speak, having their providers challenge gender and weight stereotypes, and not being accused of malingering.^31^ Positive interactions were described as those in which providers facilitate access to care by, for example, being willing to order diagnostics and make referrals to specialists.

Beyond fostering provider empathy, Côté (2024) advocates for a patient partnership approach to care, conceptualized within the Montreal Model as recognizing “patients as full-fledged team members, on an equal footing with any other professional, not only in their care team but also in governance, research, and education teams” (p. 472).^98^ This model departs from paternalistic or unilateral frameworks by incorporating principles of shared decision-making, illness self-management, and therapeutic education,^99^ while affirming the legitimacy and complementary nature of the knowledge and expertise held by all care team members, including patients themselves.^98^ The patient partnership approach seeks to enhance patient autonomy and knowledge, simultaneously restoring their political agency within healthcare systems.

## Conclusion

Findings from this study suggest that mistreatment in medical settings is predominantly systemic, rooted not only in institutional practices and professional training, but perhaps also in broader health vulnerabilities shaped by social determinants. This research illuminates a critical and potentially pervasive issue within the medical field: the insufficiency of practitioner empathy and existential care beyond courteousness, underscoring the imperative for more profound relational and structural reforms to enhance care experiences and outcomes. The present research contributes to a growing body of scholarship advocating for targeted anti-Black racism training within gynaecological and obstetrical care, emphasizing the need for culturally competent practices that also challenge cisheteronormativity and other intersecting axes of normativity and oppression.

## Supplemental material

Supplemental material - Recent obstetrical and gynaecological care experiences and their sociodemographic and mental health correlates: A latent class analysis Supplemental material for Recent obstetrical and gynaecological care experiences and their sociodemographic and mental health correlates: A latent class analysis by L.J. Séguin, S. Lévesque, A. Jean Thorn, I. Boucoiran, S. Landry and N. Godbout in Women’s Health.

## Data Availability

Since the data contain potentially sensitive information about study participants, the Université du Québec à Montréal (UQAM) Human Research Ethics Board has only approved storage of the dataset on secure institutional servers. Any requests to access the data can be made to ciereh@uqam.ca.*
